# Gastric tube resection due to metachronic cancer and a recurrence in anastomosis after Ivor-Lewis esophagectomy – case report

**DOI:** 10.1186/1477-7819-10-83

**Published:** 2012-05-16

**Authors:** Sławomir Jabłoński, Łukasz Piskorz, Marcin Wawrzycki

**Affiliations:** 1 Department of Thoracic Surgery, General and Oncological Surgery, Medical University of Lodz, 113 Żeromskiego St., 90-547, Łódź, Poland

**Keywords:** Esophageal cancer, Gastric tube cancer, Esophageal substitute, Metachronic cancer

## Abstract

Gastric tube after esophagectomy can be the site of local recurrence or the development of second primary tumor which implies poor prognosis. The study presents an extremely rare case of a patient after Ivor-Lewis esophagectomy for squamous cell carcinoma, in whom there was detected local recurrence in the anastomosis associated with metachronous primary tumor in gastric tube. Esophageal reresection with the upper part of the stomach was performed. Left colonic segment supplied by middle colic vessels transposed through retrosternal route was used as new esophageal substitute.

## Background

Esophagectomy with lymph node dissections combined with adjuvant therapy remains the basic method of esophageal cancer surgical treatment. The stomach is the most commonly used esophageal substitute after esophagectomy. Owing to the availability of new, more precise diagnostic techniques and to the improvement of the late outcomes of esophageal cancer treatment, there increases the detectability of the cases of second primary malignancies in the stomach [1]. The coexistence of squamous cell carcinoma with malignant and benign cancers of other organs (such as the head, neck, upper respiratory tract, and of the remaining part of the gastrointestinal tract) is known and concerns nearly 10% to 21% of patients [1,2]. This phenomenon even has meaningful term: ‘field cancerization’ [3]. The coexistence of primary gastric and esophageal cancer has been estimated to reach 3% to 7% [4,5]. The incidence of primary cancer within the gastric graft after esophagectomy has been estimated to be related to <2.1% of patients [4-6].

The surgical treatment of a cancer diagnosed in the gastric tube after esophagectomy is a difficult and rarely undertaken surgical challenge associated with the risk for severe complications and high mortality.

## Case report

A female patient, MZ, aged 61 years, referred primarily for surgical treatment due to esophageal cancer detected in the epicardial region. Endoscopic examination revealed a primary tumor located 30 cm from the incisors. Endoscopic evaluation of the stomach lumen was impossible due to esophageal stricture at the tumor level. Histopathological examination of tumor specimens led to the diagnosis of squamous cell carcinoma. Preoperative thoracic computed tomography (CT) detected neither tumor infiltration on the surrounding tissues nor the enlargement of the regional lymph nodes in the thorax and abdominal cavity.

Based on the results of diagnostic investigations which confirmed the possibility of local excision of the tumor, the patient was qualified for surgical treatment.

### Primary operation

Partial Ivor-Lewis esophago-gastrectomy was performed with conventional two-field en block lymphadenectomy using a gastric tube as an esophageal substitute. The esophagogastroplasty was performed in the right pleural cavity. The postoperative histopathological examination showed squamous cell carcinoma keratodes G-2 invading the adventitia without lymph node metastasis (pT_3_N_0_M_0_, Stage II A). Microscopically esophageal and gastric rings were free from neoplastic cells. The proximal resection margin was 4 cm. Eighteen lymph nodes of the following groups were removed: upper thoracic paraesophageal lymph nodes (2), right thoracic paratracheal lymph nodes (1), bifurcation lymph nodes (2), middle thoracic paraesophageal lymph nodes (2), right pulmonary hilar lymph nodes (1), lower thoracic paraesophageal lymph nodes (2), diaphragmatic lymph nodes (1), posterior mediastinal lymph nodes (2), lesser curvature lymph nodes (3), left gastric artery lymph nodes (1), and common hepatic artery lymph nodes (1). All lymph nodes were free of cancer cells. The patient’s postoperative course was uncomplicated and she was discharged from hospital after 17 days. After surgery, the patient did not report for further treatment at an oncological center.

**Figure 1:**
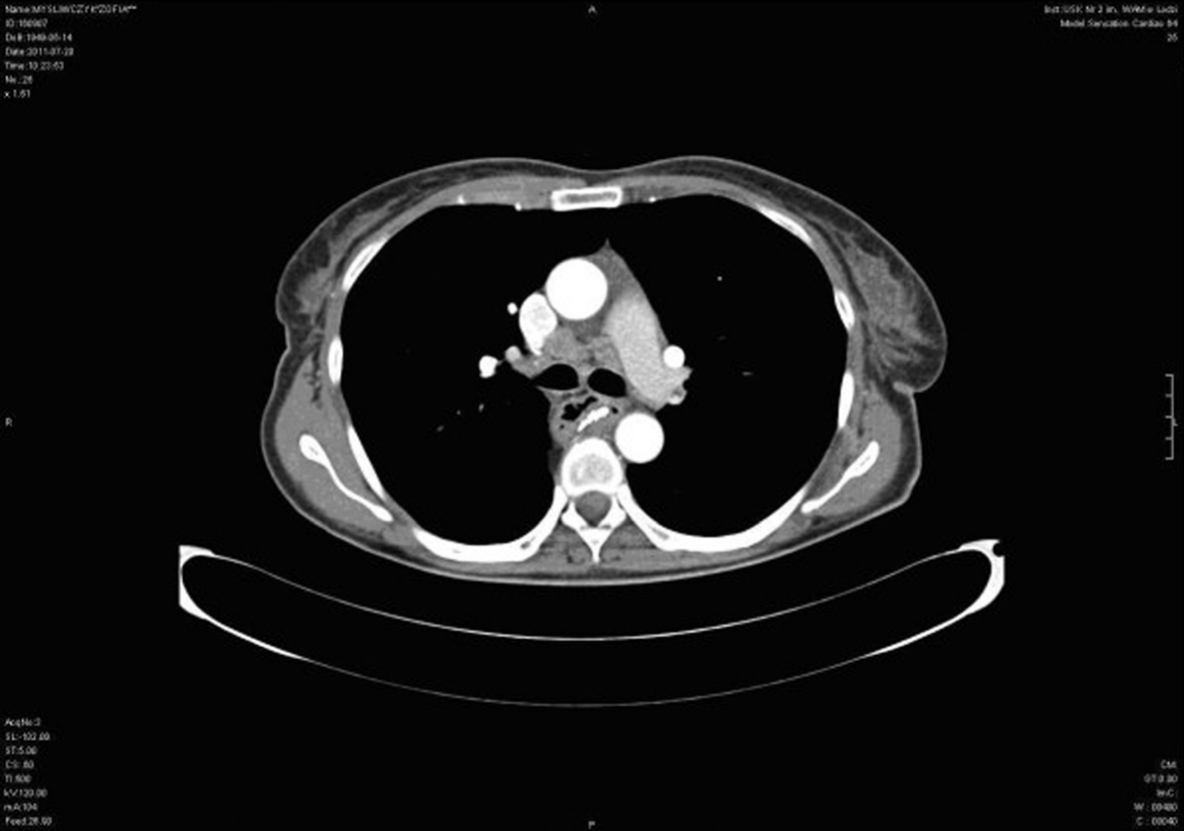
Computed tomography (scan A, B): gastroesophageal anastomosis at the level of tracheal bifurcation, below visible thickening of gastric mucosa.

The follow-up endoscopy performed 6 months after esophagectomy showed: (A) mucosa at the site of anastomosis: uneven, congested with superficial lesions covered with fibrin, unchanged esophageal mucous membrane proximal to anastomosis. About 3 cm below the anastomosis was a stocky polyp of 10 to 12 mm in diameter (B) surrounded by normal gastric mucosa. Congested mucosa without lesions were in the gastric tube distal segment. The results of histopathological examinations revealed: (A) gastric mucosa segment with the features of foveolar hyperplasia, (B) polyp from the gastric tube - squamous cell carcinoma keratodes (suspicion of local recurrence). The result of CT after esophagectomy was (Figure 1): anastomosis between the stomach and the esophagus constructed above tracheal bifurcation. No recurrence of radiological traits of the growth process were found at the anastomosis site. There was visible segmental gastric wall thickening (7 mm) about 3 to 4 cm below the anastomosis without infiltration to the surrounding structures. There were enlarged single paratracheal and subcalcarine lymph nodes. No focal lesions were observed in the lungs.

The patient was qualified for reoperation with the possibility using a colon as esophageal substitute. Earlier performed diagnostic endoscopy revealed no pathological lesions nor vascular anomalies in angio CT of abdominal vasculature.

### Surgical technique

**Figure 2:**
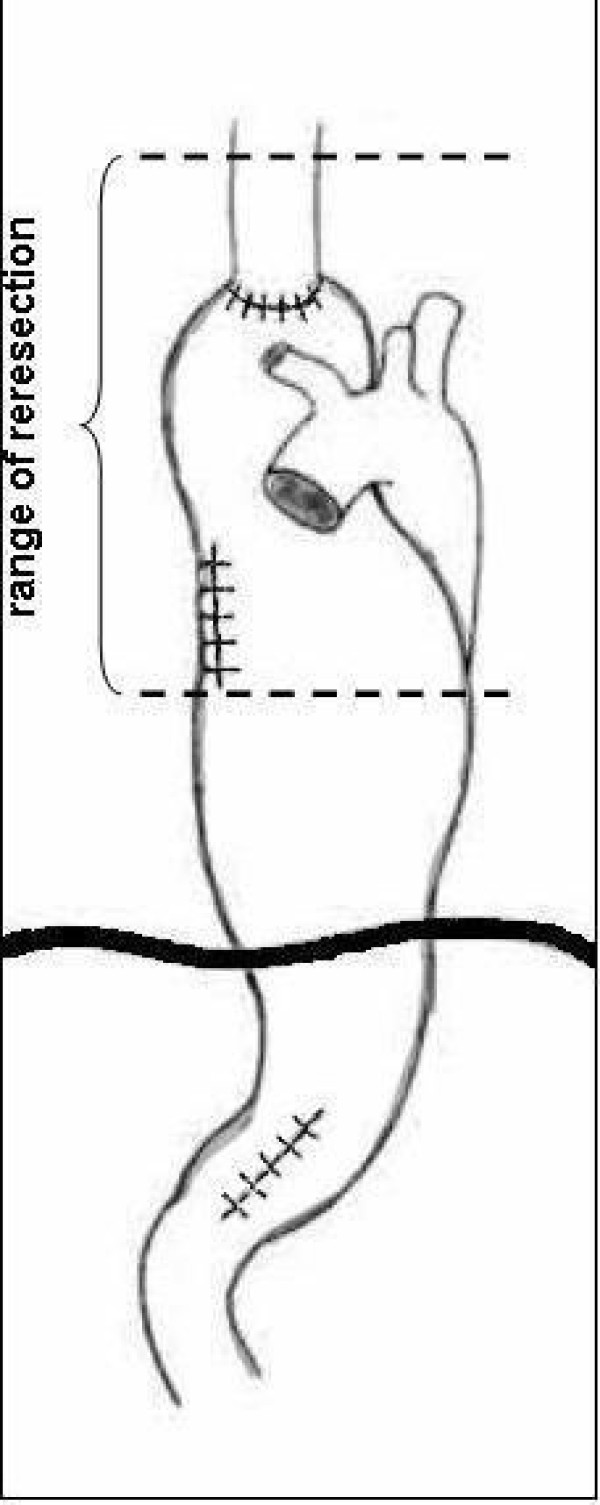
The range of gastric tube and esophageal stump resection.

**Figure 3:**
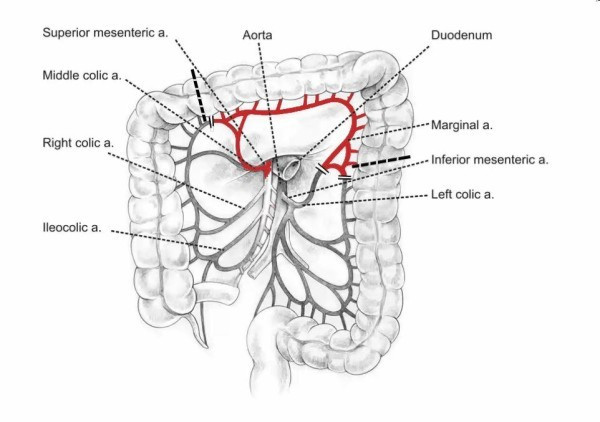
Vascularization of the long segment of transverse colon and left part of the colon on the middle colic artery.

Operation was started with right rethoracotomy to release carefully the gastric tube from adhesions with the right lung and thoracic wall. The esophageal wall thickened circumferentially about 1 to 2 cm above the anastomosis, characteristic of recurrence, was detected by intraoperative palpation (not shown on endoscopy and CT). En bloc dissection of residual esophagus was performed to the level of the apex of the chest and then the esophageal stump was cut 8 cm above the anastomosis (Figure 2). After separation of anastomoses between the gastric tube and the lung numerous air leaks from its surface were observed. Single lymph nodes were removed in the site after conduit. Gastric tube was returned back to the peritoneal cavity together with the distal part of esophageal stump. The resection of two-thirds of the proximal part of the stomach with the esophageal stump was performed through laparotomy.A new conduit was prepared from left colonic segment supplied by the middle colic artery (Figure 3). The esophageal stump was dissected from a left neck incision and limited lymphadenectomy was performed (paraesophageal and deep cervical lymph nodes). The right colon was mobilized and then pulled through the cervical incision through the retrosternal route. Hand-sewn anastomosis was made in the neck between the esophageal stump and antiperistaltic colonic loop. The distal end of conduit was anastomosed to the posterior gastric wall. Initially, jejunostomy feeding was introduced and after 5 days it was replaced with oral feeding. The total period of hospitalization was 15 days.

The results of postoperative histopathological examination were (a) distal esophageal stump with anastomosis: recurrence of squamous cell carcinoma keratodes G-2, tumor emboli in lymphatic vessels, proximal esophageal stump free of tumor infiltration; (b) tumor located in gastric tube, squamous cell carcinoma _p_T_2_N_0_Mx (primary focus), the tumor sounded by normal gastric mucosa; and (c) lymph nodes from the thoracic area (7) and neck (4) without tumor cells.

#### Postoperative course

Eight weeks after surgery, in the course of the second cycle of chemotherapy, dysphagia developed. Endoscopy revealed no anastomotic stenosis. Within a 3-week period an effective endoscopy-guided dilatation with a balloon was performed twice. Six months after reoperation no recurrence of the neoplastic process was observed in follow-up diagnostic tests.

## Discussion

The use of the stomach as an esophageal substitute after esophagectomy is commonly accepted by the majority of surgeons [4,5]. The stomach is by choice the first organ owing to such characteristics as: availability and length, plasticity, and rich submucosal vascular network [6,7]. However, part of patients can manifest the second primary tumor. The second primary carcinoma was defined according to the criteria described by Warrren and Gates [8]: (1) the tumours had to be evidently malignant on histological examination; (2) they had to be separated by normal mucosa; and (3) the possibility of a second tumor representing a metastasis had to be excluded. In the case described by us, besides the local recurrence in the anastomosis we dealt with the second primary tumor in the gastric tube which fulfilled histopathological criteria of metachronous tumour. Esophageal stenosis often makes endoscopic evaluation of the stomach impossible. In the observation of Koide [4] as much as 89.3% of gastric tumors coexisting with esophageal carcinoma were located at the upper or middle third of the stomach and nearly one-third of them were not detected before surgical treatment. We cannot exclude that due to esophageal stenosis, the secondary gastric tumor was not detected in our patient in the first endoscopy. Gastric metachronous carcinomas which are diagnosed on the basis of clinical symptoms have poor prognosis, whereas those detected early in the course of endoscopic screening have much better prognosis, particularly if they are related only to the mucosa [9,10]. Synchronous primary gastric cancers in patients with esophageal carcinoma diagnosed before esophagectomy should be treated in the same way as primary cancer [4,10]. If they are limited to mucosa, they can be resected during pre- or intraoperative endoscopy [11,12]. Other locally operative tumors can be resected during esophagectomy by performing mucosal resection using gastrotomy or adequately extending the range of gastric resection.

In the case of metachronous tumors located in the gastric tube the therapeutic management depends on the depth of tumor infiltration to the gastric wall [10-12]. In the case of second locally inoperable tumors and local recurrence the therapy is limited to chemotherapy, radiation therapy, or a combination of these modalities [13]. After esophagectomy, endoscopic mucosal resection is the therapy of choice for early gastric cancer [6,10,12]. In the selected cases of metachronous tumors limited to gastric substitute wall reconstructed retrosternally, the tumor can be resected using minimally invasive videothoracoscopic technique [14]. The surgical treatment of advanced secondary cancers in the gastric tube is a difficult and rarely undertaken challenge due to complicated local conditions, the risk of severe complications, and high mortality. Table 1 demonstrates the literature data summing up the results of the treatment of metachronous neoplastic lesions in the gastric conduit. Akiyama and Nakayama reported that in surgeries due to a cancer in the gastric tube the mortality rate could reach 50% of cases (14 of 28) [7]. In low grade tumors located in the upper third of the stomach it is suggested to perform proximal gastrectomy with the dissection of regional lymph nodes. In high grade but operative second cancers of the stomach total gastrectomy combined with esophagectomy is recommended [6,10,15]. The surgery allows removal of both tumors and complete dissection of the regional lymph nodes. According to Oki et al. the prognosis of patients who underwent resection was better than that of the other patients [15].

**Table 1:** Table summarizing published cases of metachronous neoplastic lesions in the gastric conduit

**Authors (published year)**	**Cases (*n*)**	**Surgical treatment**	**Recurrence of tumor**	**Follow-up period**	**Survival rate**
Suzuki *et al*. [11]	10	4	(NR)	(7, 42, 60, 99 m)	3 alive (NR)
Sugiura *et al*. [12]	26	10	3 cases of GC, 2 cases of EC, 4 cases of RLN	(NR)	1 alive (5 months)
Matsubara *et al*. [1]	17	NR	NR	5 years	45%
Okamoto *et al*. [10]	8	5	2	2 and 81 m	3 alive (NR)
Motoyama *et al*. [5]	2	2	No	4 and 55 m	(NR)
Yoon *et al*. (2010) [6]	10	6	1 case of EC	median 14 m (range 1-97)	70% (5 years)
Oki *et al*. [15]	10	5	2 cases (NR)	2-8 years	3 alive (NR)

EC, oesophageal cancer; GC, gastric cancer; ; m, months; NR, not reported; RLN, regional lymph node metastases.

Gastric tube resection requires reconstruction of gastrointestinal tract with the useof colonic or small intestine conduit. In such situations colon graft is preferred owing to its proper length, reliable blood supply, and fewer complications [11,12,15]. In the case described by us we decided to use retrosternal route for colon graft transposition for four reasons: the need for more extensive resection of the esophagus; the risk of locoregional recurrence; planned radiotherapy of the area of posterior mediastinum; and intensive air leak from the lung surface. Similar management was recommended by Yoon *et al.*[6]. The non-complicated postoperative course confirmed the rightness of this choice. We think that more frequent use of a colon graft as the first choice esophageal substitute is worth considering owing to lower risk of the development of second primary tumor or local recurrence at the site of esophagus removal.

## Conclusion

Gastric tube resection with the creation of a new esophageal substitute from the colon is a difficult but possible treatment option in selected cases of recurrence or second primary cancer of the stomach detected in postoperative follow-up.

## Consent

Written informed consent was obtained from the patient for publication of this report and any accompanying images**.**

## Competing interest

The authors declare that they have no competing interests in this paper.

## Authors’ contributions

SJ composed the case report, prepared and edited this manuscript, contributed it conception, collected the data and conducted a literature search. ŁP and MW participated in the data collection and gave final approval for this version of the manuscript. All authors read and approved the final manuscript.
